# Rapid Clearance of Refractory Methicillin-Resistant Staphylococcus aureus (MRSA) Bacteremia With Septic Pulmonary Emboli Following Early Ceftaroline Therapy

**DOI:** 10.7759/cureus.101645

**Published:** 2026-01-15

**Authors:** Abhinav Mahajan, Haroon Khan, Rakesh Kavuri, Asim Ruhela

**Affiliations:** 1 Internal Medicine, St. Joseph's Medical Center, Stockton, USA

**Keywords:** ceftaroline, methicillin-resistant staphylococcus aureus, refractory bacteremia, salvage therapy, septic pulmonary emboli

## Abstract

Methicillin-resistant *Staphylococcus aureus* (MRSA) bacteremia is associated with significant morbidity and mortality, particularly when persistent despite appropriate therapy. Refractory bacteremia often reflects inadequate source control, metastatic infection, or antimicrobial treatment failure and requires timely reassessment and escalation of care.

We report the case of a 44-year-old male patient with poorly controlled type 2 diabetes mellitus and polysubstance use who presented with altered mental status and was found to have diabetic ketoacidosis, necrotizing soft tissue infection of the right lower extremity, and MRSA bacteremia. His hospital course was complicated by septic pulmonary emboli and lung abscesses. Despite prompt initiation of intravenous vancomycin, therapeutic drug levels, and definitive surgical source control via below-knee amputation, surveillance blood cultures remained persistently positive for MRSA. Following multidisciplinary evaluation, vancomycin was discontinued and intravenous ceftaroline was initiated, resulting in rapid clearance of bacteremia and sustained clinical improvement. The patient was discharged to a skilled nursing facility to complete a six-week course of ceftaroline, with no evidence of recurrent infection on follow-up.

This case underscores the importance of early recognition of vancomycin failure in persistent MRSA bacteremia and highlights the role of ceftaroline as an effective salvage therapy, particularly in patients with metastatic infection and high-risk comorbidities. Multidisciplinary management and individualized antimicrobial strategies remain critical for achieving favorable outcomes in refractory MRSA infections.

## Introduction

*Staphylococcus aureus* bacteremia (SAB), particularly methicillin-resistant strains, remains a major cause of severe infection worldwide [1]. Despite widespread use of vancomycin as first-line therapy, persistent or refractory methicillin-resistant *Staphylococcus aureus* (MRSA) bacteremia continues to occur and is associated with high mortality, reported at approximately 20%-30%, as well as complications including septic emboli, endocarditis, osteomyelitis, and abscess formation [2,3].

Evaluation of SAB requires a thorough history and physical examination to identify the primary source, such as skin and soft tissue infection, intravenous drug use, indwelling vascular devices, or prosthetic material, and to assess for metastatic foci, including osteoarticular, cutaneous, or cardiac involvement. Recommended diagnostic evaluation includes early infectious disease consultation, echocardiography, and serial follow-up blood cultures to confirm clearance of bacteremia [2].

Vancomycin remains the cornerstone of MRSA therapy; however, treatment failure and persistent bacteremia are increasingly reported. Studies have described persistent bacteremia rates of approximately 5%-20%, commonly defined as bacteremia lasting longer than four days despite appropriate therapy [4]. In addition, nephrotoxicity remains a significant concern, particularly when vancomycin is used in conjunction with other nephrotoxic agents [5,6]. Mechanisms contributing to vancomycin failure include suboptimal tissue penetration, high bacterial inoculum with biofilm formation, and altered pharmacokinetics in critically ill patients [6,7].

Refractory MRSA bacteremia poses substantial therapeutic challenges and often necessitates alternative or combination antimicrobial strategies. Ceftaroline, a fifth-generation cephalosporin with potent anti-MRSA activity, is FDA-approved for acute bacterial skin and soft tissue infections and community-acquired bacterial pneumonia. Although not formally approved for MRSA bacteremia, growing evidence supports its use as salvage therapy in complicated MRSA infections, including endocarditis and persistent bacteremia [8,9].

A large systematic review and meta-analysis of more than 500,000 cases published between 2011 and 2021 reported an estimated three-month mortality of approximately 30%, although mortality has gradually declined with standardized management and improvements in quality of care [10,11].

We report a case of refractory MRSA bacteremia in a patient with poorly controlled diabetes mellitus and methamphetamine use, complicated by necrotizing fasciitis requiring below-knee amputation, septic pulmonary emboli, and lung abscess formation. Despite appropriate vancomycin therapy and definitive surgical source control, bacteremia persisted and ultimately resolved following transition to ceftaroline, underscoring the importance of early recognition of vancomycin failure and timely consideration of alternative antimicrobial therapies.

## Case presentation

A 44-year-old male patient with a history of type 2 diabetes mellitus, hypertension, and polysubstance abuse presented after being found altered and confused on the street. Paramedics reported a blood glucose level greater than 600 mg/dL. The patient endorsed chest pain, shortness of breath, and right foot pain.

On arrival to the emergency department, he was lethargic but arousable, tachycardic with a heart rate of 130-140 beats per minute, tachypneic with a respiratory rate of 30-40 breaths per minute, normotensive with a blood pressure of 130/70 mmHg, and saturating well on room air. Physical examination revealed a necrotic, malodorous right foot wound with surrounding erythema, swelling, and palpable crepitus.

The patient had a history of chronic right foot wounds with multiple prior musculoskeletal infections, including septic arthritis and osteomyelitis, as well as prior orthopedic interventions, including right ankle fixation and partial ray amputation of the right fourth and fifth metatarsals. Poor follow-up and recurrent infections placed him at increased risk for severe and progressive infection.

Initial laboratory evaluation demonstrated leukocytosis, anemia, severe hyperglycemia, high anion gap metabolic acidosis consistent with diabetic ketoacidosis, acute kidney injury, and markedly elevated inflammatory markers. Admission laboratory findings are summarized in Table 1.

**Table 1:** Laboratory findings on admission.

Laboratory test	Result	Reference range	Interpretation
White blood cell count (WBC)	21.5 K/µL	4.0-11.0 K/µL	Leukocytosis, consistent with acute infection/sepsis
Hemoglobin	10.7 g/dL	13.5-17.5 g/dL	Anemia
Sodium	124 mmol/L	135-145 mmol/L	Hyponatremia
Corrected sodium	132 mmol/L	135-145 mmol/L	Partially corrected hyponatremia due to hyperglycemia
Blood urea nitrogen (BUN)	32.9 mg/dL	7-20 mg/dL	Elevated, consistent with acute kidney injury
Creatinine	2.0 mg/dL	0.7-1.3 mg/dL	Acute kidney injury
Estimated GFR	41 mL/min/1.73 m²	>60 mL/min/1.73 m²	Reduced renal function
Serum glucose	610 mg/dL	70-110 mg/dL	Severe hyperglycemia
Hemoglobin A1c	13.20%	<5.7%	Poorly controlled diabetes mellitus
Serum bicarbonate	6 mmol/L	22–28 mmol/L	Severe metabolic acidosis
Anion gap	30	8–16	Elevated, consistent with high anion gap acidosis
β-hydroxybutyrate	15.87 mmol/L	<0.4 mmol/L	Markedly elevated, diagnostic of diabetic ketoacidosis
Arterial pH	7.22	7.35-7.45	Acidemia
Arterial pCO₂	14 mmHg	35-45 mmHg	Respiratory compensation
Arterial HCO₃⁻	5.7 mmol/L	22-26 mmol/L	Severe metabolic acidosis
C-reactive protein (CRP)	40 mg/L	<5 mg/L	Markedly elevated inflammatory marker
Erythrocyte sedimentation rate (ESR)	>130 mm/hr	<20 mm/hr	Markedly elevated inflammatory marker
Urine toxicology	Positive for methamphetamine/amphetamine	Negative	Consistent with polysubstance use

**Figure 1:**
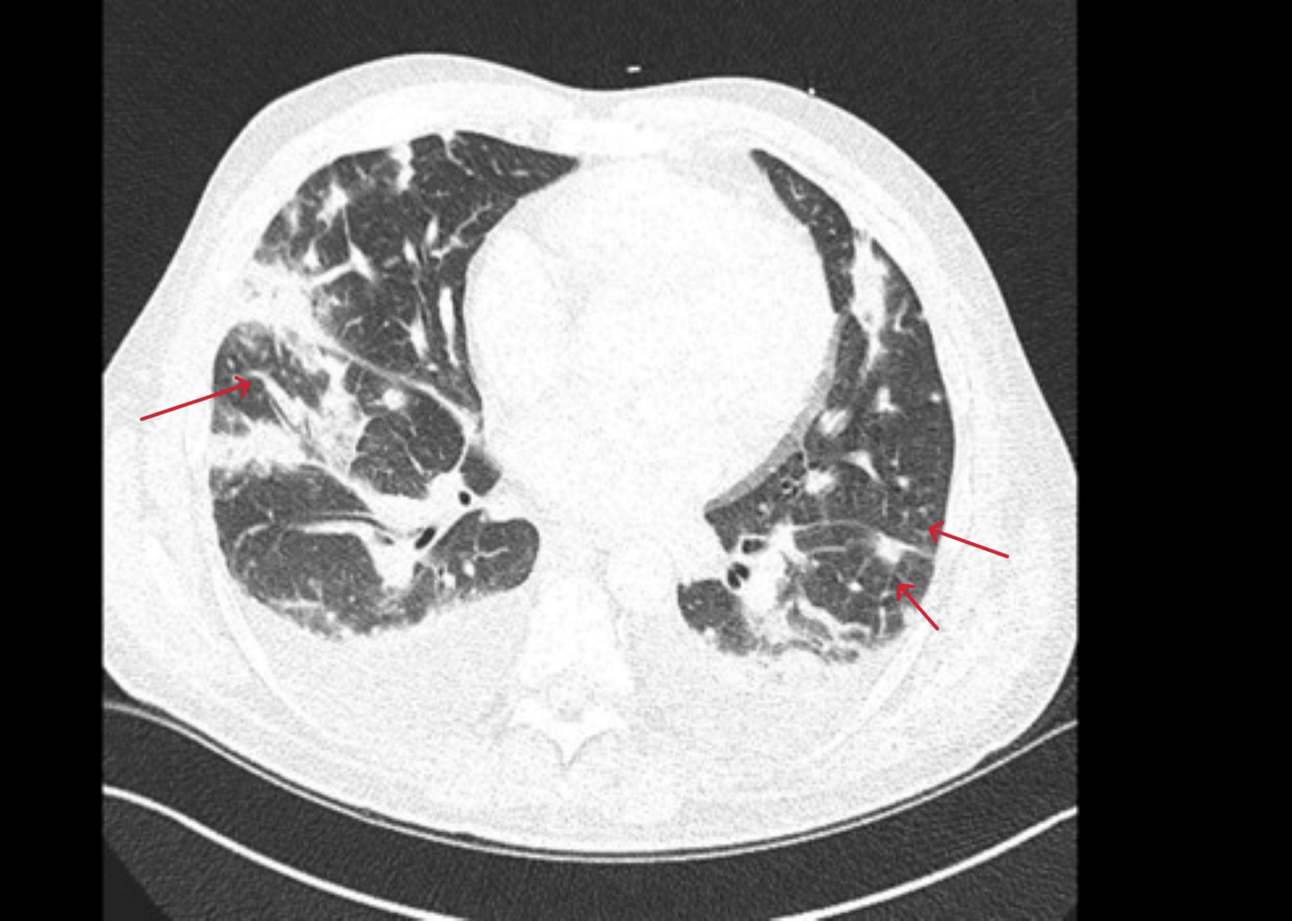
Axial computed tomography angiography of the chest demonstrating multiple bilateral peripheral cavitary pulmonary nodules (arrows), consistent with septic pulmonary emboli in the setting of methicillin-resistant Staphylococcus aureus bacteremia.

**Figure 2:**
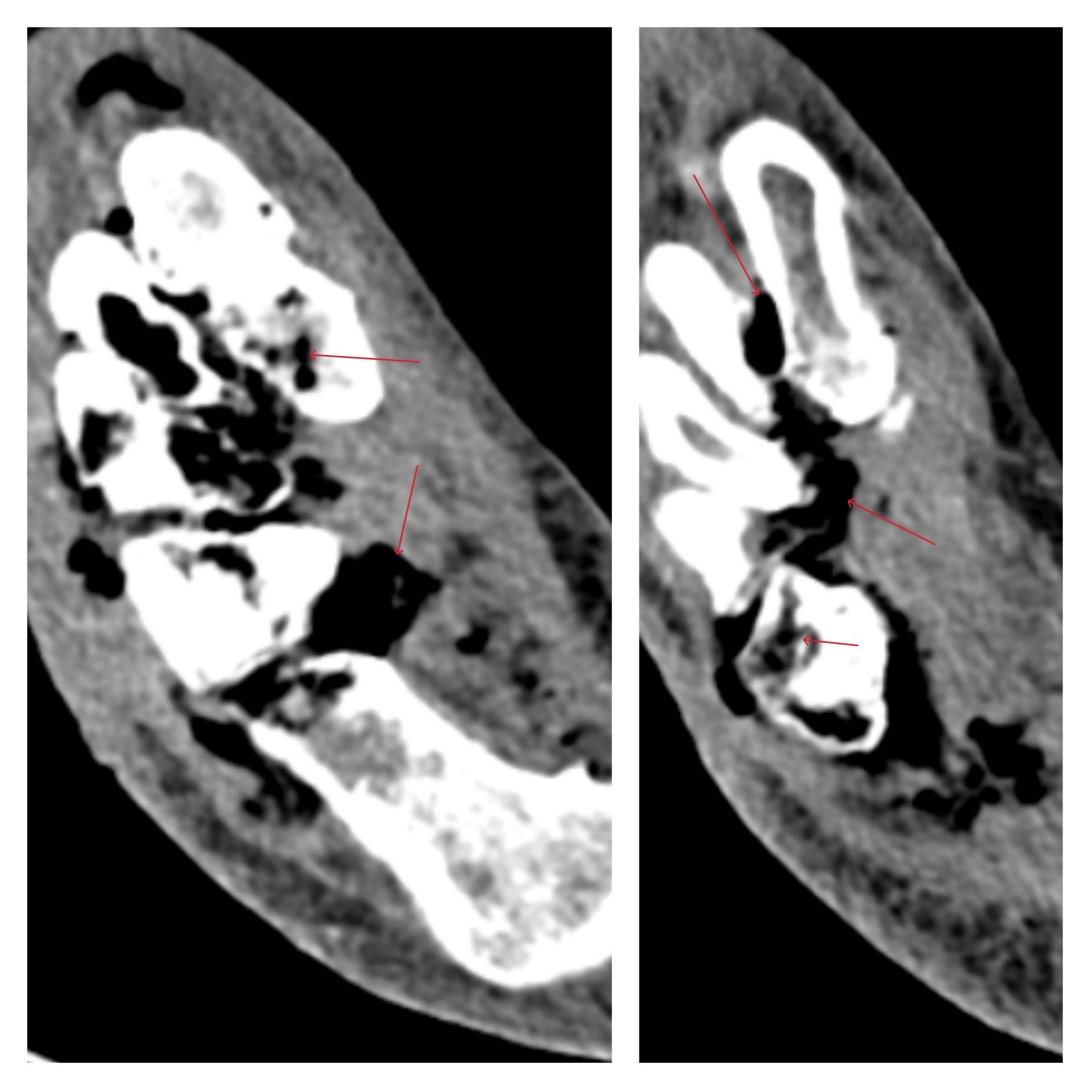
Axial contrast-enhanced computed tomography images of the right foot demonstrating extensive soft tissue gas tracking along the fascial planes (arrows), with associated soft tissue edema and osseous involvement, consistent with necrotizing soft tissue infection and underlying osteomyelitis.

**Figure 3:**
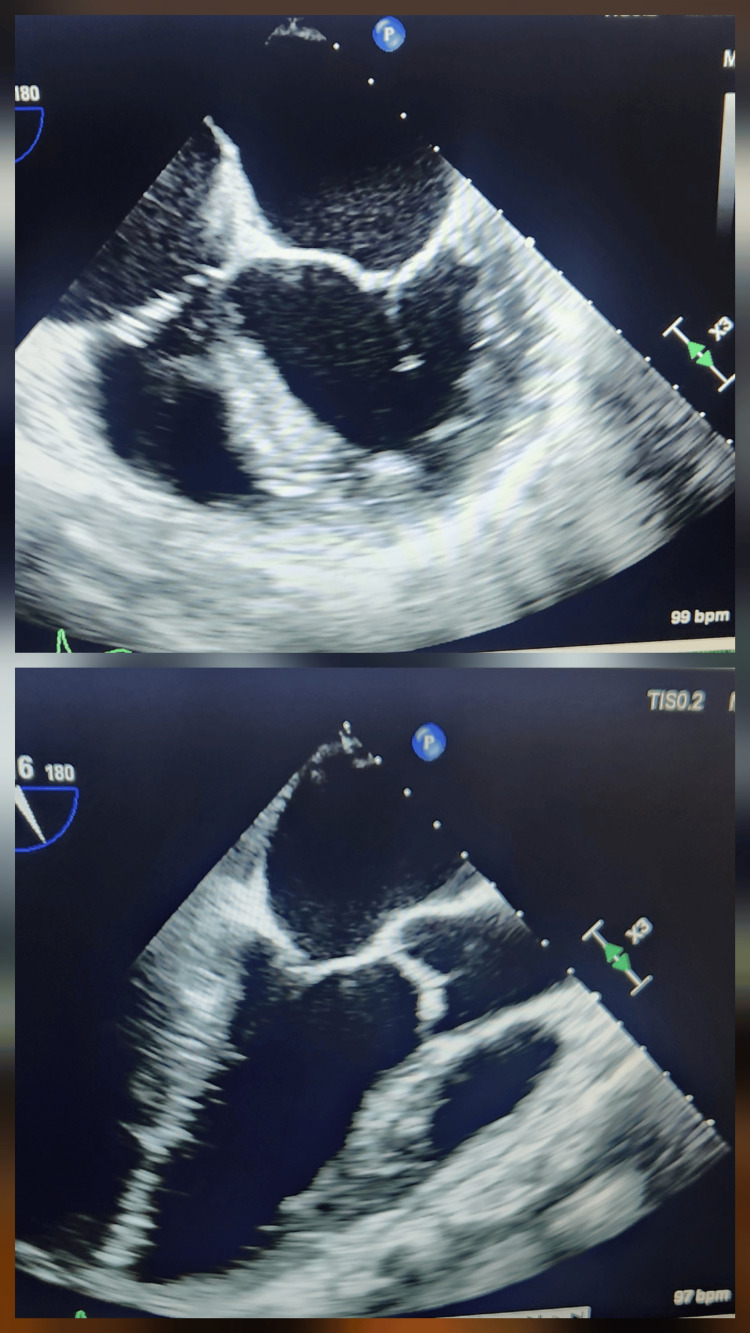
Transesophageal echocardiography demonstrating normal mitral and tricuspid valves, with no evidence of valvular vegetations or intracardiac thrombus, effectively excluding infective endocarditis in the setting of persistent methicillin-resistant Staphylococcus aureus bacteremia.

Computed tomography (CT) angiography of the chest revealed multiple cavitary pulmonary nodules throughout both lungs, consistent with septic emboli or metastatic infection (Figure 1). CT of the right lower extremity with contrast demonstrated necrotizing soft tissue infection involving the right foot and ankle with underlying osteomyelitis and extensive soft tissue gas (Figure 2). Transthoracic and subsequent transesophageal echocardiography showed no valvular vegetations or intracardiac thrombus (Figure 3). Vascular studies demonstrated flow-limiting stenosis of the distal right posterior tibial artery without evidence of deep vein thrombosis; representative images were not available for inclusion.

Blood cultures obtained on admission grew MRSA in four out of four bottles. Superficial wound cultures grew *Morganella morganii*, *Enterococcus *species, and *S. aureus*. The patient was initiated on intravenous vancomycin 1000 mg every 12 hours and underwent urgent right below-knee amputation for necrotizing fasciitis. Surgical pathology confirmed gangrenous tissue with acute and chronic osteomyelitis. Despite six days of intravenous vancomycin with therapeutic trough levels, repeat blood cultures obtained on hospital days 2 and 4 remained positive, and the patient continued to have intermittent fevers.

Given persistent MRSA bacteremia despite definitive surgical source control, a multidisciplinary team including infectious disease, orthopedic surgery, pulmonology, and pharmacy initiated intravenous ceftaroline 600 mg every 12 hours on hospital day 6 for a planned six-week course, and vancomycin was discontinued. Subsequent blood cultures cleared, with no growth detected by hospital day 11. The patient remained clinically stable, afebrile, and maintained oxygen saturation greater than 93% on low-flow supplemental oxygen. He was discharged to a skilled nursing facility to complete a six-week course of intravenous ceftaroline and to continue rehabilitation following below-knee amputation. Follow-up confirmed adherence to antimicrobial therapy and absence of recurrent infectious symptoms.

The final diagnosis was sepsis secondary to necrotizing soft tissue infection of the right lower extremity, complicated by refractory MRSA bacteremia and pulmonary septic emboli, in the setting of poorly controlled diabetes mellitus and polysubstance use.

## Discussion

MRSA bacteremia remains a major contributor to morbidity and mortality, with reported mortality rates ranging from 20% to 30% despite appropriate therapy [1,2]. Persistent or refractory bacteremia, as seen in this patient, represents a particularly challenging clinical scenario. Refractory MRSA bacteremia is frequently associated with metastatic infections, including septic pulmonary emboli, osteomyelitis, and endocarditis, or with inadequate source control [3,4].

In this case, persistent MRSA bacteremia occurred despite initial vancomycin therapy and urgent surgical source control. Several mechanisms may explain vancomycin failure, including suboptimal tissue penetration, particularly in necrotic tissue or abscess cavities, high bacterial inoculum with biofilm formation, and altered pharmacokinetics in critically ill patients, leading to subtherapeutic exposure despite standard dosing [5,6]. Host-related factors such as poorly controlled diabetes, polysubstance use, and chronic musculoskeletal infection further predispose patients to severe infection and reduced treatment response [2,4]. Methamphetamine use further exacerbates infection risk by causing vasoconstriction, tissue ischemia, impaired wound healing, and immune dysregulation, as well as increasing the likelihood of skin breakdown and injection-related or traumatic inoculation of pathogens. Together, these factors created a high-risk environment for invasive MRSA infection of the right lower extremity.

Pulmonary septic emboli likely served as a secondary reservoir for persistent bacteremia. Septic emboli arise from infected thrombi or metastatic foci and may progress to cavitation, lung abscess formation, or pleural involvement [3,7]. Identification and monitoring of these metastatic sites are critical, as persistent bacteremia often continues until all infectious foci are adequately addressed [3,4]. In this patient, serial imaging demonstrated interval progression of pulmonary nodules, underscoring the importance of ongoing surveillance when bacteremia fails to clear [3].

Given vancomycin failure, ceftaroline was initiated, resulting in blood culture clearance within days. Ceftaroline is a fifth-generation cephalosporin with potent activity against MRSA, and emerging evidence supports its use as salvage therapy in refractory MRSA bacteremia, particularly when first-line therapy fails despite adequate source control [7-10]. Multiple studies have reported favorable microbiologic and clinical outcomes, even in patients with metastatic infection and significant comorbidities [7-10].

This case also highlights the importance of multidisciplinary care. Collaboration among infectious disease, orthopedic surgery, podiatry, and rehabilitation teams facilitated timely source control, optimization of antimicrobial therapy, and coordinated postoperative management [3,4]. Early interdisciplinary involvement and frequent reassessment were instrumental in preventing further complications and supporting recovery.

In summary, this case illustrates the complex interplay between host factors, microbial virulence, and antimicrobial limitations in refractory MRSA bacteremia. It emphasizes the importance of early recognition of treatment failure, comprehensive evaluation for metastatic infection, and timely initiation of salvage therapy within a multidisciplinary care framework [2-10].

## Conclusions

Refractory MRSA bacteremia remains a serious and complex clinical challenge, particularly in patients with significant comorbidities and metastatic infection. This case highlights the importance of early recognition of treatment failure, thorough evaluation for persistent infectious foci, and prompt escalation of antimicrobial therapy when first-line agents are ineffective. Despite therapeutic vancomycin dosing and definitive surgical source control, bacteremia persisted in this patient until therapy was transitioned to ceftaroline, resulting in sustained microbiologic clearance and clinical improvement. Ceftaroline should be considered a viable salvage option in cases of persistent MRSA bacteremia, especially when complicated by metastatic infection and host-related risk factors. A multidisciplinary approach and individualized treatment strategy are essential to optimizing outcomes in these high-risk patients.
